# Designing a 2000-Calorie Balanced Indian Diet for Diabetic Adults: Controlled Fat Intake and 50% Energy From Carbohydrates

**DOI:** 10.7759/cureus.89449

**Published:** 2025-08-05

**Authors:** Vasudevan P Sankaran, D M Vasudevan

**Affiliations:** 1 Pathology, Govt. Medical College, Thrissur, IND; 2 Research, Jubilee Mission Medical College and Research Institute, Thrissur, IND

**Keywords:** 2000 calories, balanced diet, balanced fat, dietary fat consumption, omega-6/omega-3 ratio

## Abstract

Setting: The study was planned with the objective of developing a balanced diet menu based on Indian traditional food items, incorporating formatted fat and 50% of energy from carbohydrates, in line with general dietary recommendations. This was undertaken in the absence of any clear, viable, or India-specific menus or guidelines for constructing such a diet. The fat formatting, guided by metabolic expert recommendations and derived from routine/local foods, adds a unique aspect to the newly formulated menu.

Objective: This study aimed to demonstrate the feasibility of designing a complete balanced diet of 2000 calories, providing 50% of energy from carbohydrates, using commonly consumed Indian food items. The menu also aims to balance fat intake by providing equal amounts of saturated and unsaturated fats.

Design: The methodology involved screening 592 locally available Indian food items for their nutritional values. A sample menu table was then constructed to serve as a prototype for a balanced diet. Nutrient data were sourced from the National Institute of Nutrition (Hyderabad, India), the USDA nutritional database, and other standard references.

Results: It was possible to formulate a balanced diet menu with mathematically derived estimates for energy contribution, fat composition, and other nutrient values. The resulting menu was deficient in only four nutrients, sodium, chloride, iodine, and fluoride, which are not expected to be adequately supplied by the chosen food items alone (e.g., sodium and chloride from table salt, iodine from iodized salt or marine foods, and fluoride from drinking water).

Conclusions: Although predictive, the model and its methodology provide a practical framework for designing metabolically targeted diets for various health conditions, with minimal resource waste. The included tables can also serve as tools for assessing nutritional content both before and after cooking. Importantly, the proposed menu’s use of familiar and affordable food items may enhance acceptability and feasibility among Indian populations.

## Introduction

Although most advanced reference books on internal medicine do not specify the daily recommended amounts of carbohydrates, proteins, and fats for individuals with diabetes, some published studies have reported improvements in glycemic control among those following the traditional Mediterranean diet (TMD) or the American Diabetes Association (ADA) diet, both of which derive 50.00%-55.00% of total energy from carbohydrates [1-5]. However, sample diets from these categories often feature foods commonly found in the Mediterranean region or Western countries, which may be scarce or expensive in India. To address this gap, the authors developed a prototype menu designed to provide 50.00% of total energy from carbohydrates using rice and other routinely used food items commonly found in Indian households, specifically for diabetic Indian adults. Key features of the proposed menu include rice and wheat as the primary cereals, milk as the main source of first-class protein, and a balanced fat profile, equal proportions of saturated fat (SAFA), monounsaturated fat (MUFA), and polyunsaturated fat (PUFA), with an omega-6 to omega-3 fatty acid ratio of 4:1 [6-11]. The technical value of this model lies in its capacity to provide mathematically derived predictions of nutritional quality, enabling easy adaptation for metabolically targeted diets for other health conditions. Socioeconomically, the model is likely to gain wide acceptance in the Indian context, owing to its reliance on familiar ingredients and minimal cost. As an alternative to established models like the TMD and ADA diets, and composed of culturally familiar food items, the new formulation may be easier to clinically evaluate, potentially bypassing the need for extensive xenobiotic assessments, such as toxicological testing or animal trials.

## Materials and methods

The general methodology involved screening 592 widely available food items in India and organizing them into columns (items) and rows (nutritive values) for further calculation and analysis.

Initial steps

The first step in designing a menu to derive 50.00% of total energy (i.e., 1,000.00 out of 2,000.00 calories) from carbohydrates, while adhering to traditional food habits, involved the initial selection of 250.00 g of rice (since 1 g of carbohydrate provides 4 kcal, 1,000 ÷ 4 = 250). This was later adjusted to 130.00 g of rice, along with the addition of 50.00 g of whole wheat flour (atta), for reasons explained below [12]. Given that diabetes mellitus is a cellular metabolic disorder characterized by impaired energy extraction from carbohydrates, the caloric contribution from protein, requiring higher energy expenditure for metabolism, was limited to 14.00% (i.e., 280.00 calories), which lies at the upper end of the normal range. Consequently, fat was set at 36.00% of total energy, considering it to be the easiest macronutrient to metabolize with the lowest specific dynamic action.

To meet the protein requirement (280 ÷ 4 = 70.00 g), 70.00 g of cowpea seeds were chosen for their traditional use and availability as a protein-rich pulse. However, rice, wheat, and cowpeas are not pure macronutrient sources. For instance, per 100.00 g, rice contains 77.16 g carbohydrates (excluding 3.74 g fiber) and 7.81 g protein; wheat contains 64.17 g carbohydrates (excluding 11.36 g fiber) and 10.57 g protein; and cowpeas contain 49.40 g carbohydrates (excluding 10.60 g fiber) and 23.50 g protein [13]. Thus, the combined cereal-pulse selection yielded only 166.97 g of carbohydrates and 31.89 g of protein, falling short of the targeted 250.00 g and 70.00 g, respectively, leaving daily deficits of 83.03 g of carbohydrate and 38.11 g of protein. These deficits were evaluated and found to be appropriate for being fulfilled by additional food items yet to be added (such as protective foods for vitamins and minerals, and fat sources). This justified both the revised quantity of rice and the inclusion of whole wheat flour (Table 1, Block A: I; Supplemental material 1).

**Table 1:** Carbohydrate, protein, fat, and dietary fiber content of specific quantities of selected food items. CHO: carbohydrate, SAFA: saturated fatty acid, MUFA: monounsaturated fatty acid, PUFA: polyunsaturated fatty acid, O_6_: omega-6 acid, O_3_ omega-3 acid. ^†^Glycemic index: wheat whole flour, 68; arrowroot flour, 65. Source: [3-18].

Food items	Block A			Block B		
	CHO (g) (target 250.00 g, excluding fiber)	Protein (g) (target 70.00 g)	Fiber (g) (target 38.00 g)	SAFA (g)	MUFA (g)	PUFA (g)
I. Cereal-pulse block				I. The initial fat combination		
1. Rice, parboiled, milled 130.00 g	100.31	7.48	0.30	0.17	0.43	100.31
2. Wheat whole flour (atta) 50.00 g^†^	32.08	5.29	5.68	0.10	0.07	0.37
3. Raw cowpea seeds 70.00 g	34.58	16.45	7.42	0.23	0.07	0.38
Total: CHO, protein, and fiber	166.97	31.89	17.96			
Inherent CHO and protein deficits of the cereal-pulse combination	-83.03	-38.11				
II. Fat/oil block						
4. Sesame oil 10.00 g				1.42	3.97	4.17
5. Rice bran oil 34.00 g				7.00	13.36	11.90
6. Rice bran 20.50 g	5.88	2.75	4.31	0.85	1.55	1.53
7. Flaxseed 22.50 g (9.00 tsp powder)	0.36	4.12	6.14	0.82	1.69	6.46 (O_3_: 5.13)
8. Coconut oil 5.36 g (as 16.00 g of meat)	0.99	0.53	1.44	4.65	0.23	0.06
9. Milk 600.00 g	28.80	18.90	0.00	11.16	4.86	1.20
10. Fenugreek seeds 12.50 g	1.32	3.18	5.94	0.10	0.09	0.39 (O_3_: 0.14)
11. Olive oil 1.25 g				0.17	0.91	0.13
Totals	37.35	29.48	17.83	26.70	26.91	26.87
The remaining inherent CHO and protein deficits of the cereal-pulse combination	-45.68	-8.63				
III. Protectives’ block				II. Details of fats from the protective food items		
12. Indian gooseberry 15.00 g	2.06	0.08	0.51			
13. Arrowroot flour 35.50 g^†^	30.10	0.11	1.21			
14. Onion stalk 125.00 g	3.11	2.33	6.38			
15. Chili powder 6.00 g	1.77	0.76	1.87	0.07	0.43	0.20 (O_6_: 0.18; O_3_: 0.01)
16. Drumstick leaves 60.00 g	3.37	3.85	4.93	0.34	0.06	0.40 (O_6_: 0.13; O_3_: 0.27)
17. Ash gourd 50.00 g	1.42	0.40	1.69			
18. Ginger, fresh 5.00 g	0.45	0.11	0.27			
19. Guavas, common, raw 40.00 g	3.56	1.02	2.16			
Totals	45.84	8.66	19.02	0.41	0.49	0.60 (O_6_: 0.31; O_3_: 0.28)
The final nominal excess amounts of CHO and protein	+0.16	+0.03				
Difference: target - total	250.00 – 250.16 = +0.16 nominal excess	70.00 – 70.03 = +0.03 nominal excess	Grand total = 54.81	Total of SAFA, MUFA, and PUFA = 1.50		

Interim nutritive evaluation 1

The cereal-pulse combination provided 2.37 g of fat, along with essential amino acids such as histidine, tryptophan, threonine, and the phenylalanine-tyrosine pair, in the required amounts. Additionally, it supplied adequate levels of folate, iron, copper, chromium, molybdenum, and manganese (Table 2; Supplemental materials 1 and 2).

**Table 2:** Protective nutrients and essential amino acids provided by the cereal-pulse combination (130 g rice, 50 g wheat, and 70 g raw cowpea seeds). -No data available on the detectable amount available in the specified quantity.

Fat-soluble vitamins				Water-soluble vitamins								Minerals and trace elements															Essential amino acids (adult male, 70 kg)										
																																						Nutrients	Vitamin A (IU or mcg)	Vitamin D (mcg)	Vitamin E (mg)	Vitamin K (mcg)	Thiamin (B1) (mg)	Riboflavin (B2) (mg)	Niacin (B3) (mg)	Pyridoxine (B6) (mg)	Pantothenic acid (B5) (mg)	Folate (B9) (mcg)	Vitamin B12 (mcg)	Vitamin C (mg)	Calcium (mg)	Phosphorus (mg)	Magnesium (mg)	Sodium (mg)	Potassium (mg)	Iron (mg)	Iodine (mcg)	Fluoride (mg)	Zinc (mg)	Copper (mcg)	Chromium (mcg)	Selenium (mcg)	Molybdenum (mcg)	Manganese (mg)	Chloride (g)	Histidine (mg)	Isoleucine (mg)	Leucine (mg)	Lysine (mg)	Methionine (mg)	Phenylalanine (mg)	Tyrosine (mg)	Threonine (mg)	Tryptophan (mg)	Valine (mg)
Adult: recommended dietary allowance (RDA), adequate intake (AI), or estimated average requirement (EAR) per day	RDA: 900 mcg (beta-carotene 3600 mcg)	RDA: 15	RDA: 15	AI: 120	RDA: 1.2	RDA: 1.3	RDA: 16	RDA: 1.3	AI: 5.0	RDA: 400	RDA: 2.4	RDA: 90	RDA: 1,000	RDA: 700	RDA: 400	AI: 1,500	AI: 3,400	RDA: 8.0	RDA: 150	AI: 4.0	RDA: 11	RDA: 900	AI: 35	RDA: 55	RDA: 45	AI: 2.3	AI: 2.3	ER: 700	ER: 1,400	ER: 2,730	ER: 2,100	ER: 700	Combined ER: 1,750		ER: 1,050	ER: 280	ER: 1,820
Food items																																					
Rice 130.00 g	0	0	0.08	1.95	0.22	0.08	3.26	0.29	0.72	12.68	0	0	10.54	182	34.74	4.11	184.6	0.94	0	0	1.4	351	6.5	1.55	71.5	1.03	_	238.59	420.34	820.37	347.23	251.8	521.86	449.77	328.95	116.75	635.58
Wheat flour (atta) 50.00 g	1.34	6.72	0.11	0.75	0.21	0.08	1.19	0.13	0.44	14.61	0	0	15.47	157.5	62.5	1.02	155.5	2.05	0	0	1.43	240	3	26.56	11	1.49	-	135.3	199.78	323.97	128	93.5	265.84	111	136.36	52.32	270.59
Raw cowpea seeds 70.00 g	21	0	0.27	3.5	0.6	0.16	1.46	0.25	1.05	443.3	0	1.05	77	296.8	128.8	11.2	777	5.79	0	0	2.36	591.5	70	6.3	70	1.07	_	511	669.2	1260	1113	234.5	959	532	626.5	203	784
Totals	Beta-carotene 22.34	6.72	0.46	6.2	1.03	0.32	5.91	0.67	2.21	470.59	0	1.05	103.01	636.3	226.04	16.33	1117.1	8.78	0	0	5.19	1182.5	79.5	34.41	152.5	3.59	_	884.89	1289.3	2404.3	1588.2	579.8	1746.7	1092.8	1091.8	372.07	1690.2
Totals (in percentage of requirement)	0.62%	44.80%	3.07%	5.17%	85.83%	24.60%	36.94%	51.54%	44.20%	117.65%	0.00%	1.17%	10.30%	90.90%	56.51%	1.09%	32.86%	109.75%	0.00%	0.00%	47.18%	131.39%	227.10%	62.56%	338.89%	156.50%	_	126.41%	92.09%	88.07%	75.63%	82.83%	162.26%		103.98%	132.88%	92.87%
Remarks	Stored nutrient	Stored nutrient	Stored nutrient	Stored nutrient						Adequate	Stored nutrient					4.4 g NaCl provides adequate amount		Adequate	Tissue-stored nutrient	In India, water levels are adequate		Adequate	Adequate		Adequate	Adequate	4.4 g NaCl provides adequate amount	Adequate				Combined ER of methionine + cysteine = 1050.00	Adequate for the combined ER of 1750.00		Adequate	Adequate	

For the second step of the workup, since no natural dietary fat sources were found to contain equal amounts of saturated (SAFA), monounsaturated (MUFA), and polyunsaturated fats (PUFA) with an internal omega-6 to omega-3 ratio of 4:1, the authors attempted to formulate a suitable combination of fat-rich foods (“fat combination”) that would meet these criteria as part of the menu.

To fulfill the remaining 36.00% of energy from fat (720.00 calories), an initial quota of 80.00 g of oils was tried (i.e., 720 ÷ 9 = 80), empirically selecting one predominant source for each fat type: coconut oil (SAFA), rice bran oil (MUFA), and sesame oil (PUFA), all locally available. However, using only these oils failed to address the carbohydrate and protein deficits inherent to the cereal-pulse combination. Thus, the trials were repeated with added oilseeds and milk. The final combination (Table 1, Block A: II; Table 3; Supplemental Material 3) included sesame oil, rice bran oil, rice bran, flaxseed, coconut, milk, fenugreek seeds, and olive oil [6,12,14-19]. Together, these items contributed approximately 37.00 g of carbohydrate and 30.00 g of protein, reducing the deficits from -83.03 g to -45.68 g for carbohydrates, and from -38.11 g to -8.63 g for proteins (Table 1, Block A: II). Notably, the protein from flaxseed and milk was of first-class quality, being rich in essential amino acids.

**Table 3:** Dietary fat source combination providing equal proportions of saturated, monounsaturated, and polyunsaturated fatty acids with a 4:1 omega-6 to omega-3 ratio in the polyunsaturated component. SAFA: saturated fatty acid, MUFA: monounsaturated fatty acid, PUFA: polyunsaturated fatty acids. The total amount of rice bran oil is 38.25 g (items 5 + 6). ^†^20.00 g coconut meat = 6.70 g of coconut oil (here as 16.00 g of fresh meat) ^‡^Regarding the totals, variations in the total values of the bottom row (around 26.70 g, within a ±0.30 g range) are due to the unique natural composition of the oils used and the rounding of their quantities to whole numbers or 0.25 g increments. Source:  [6,12-19].

Food items (g)	Fatty acids (g)		
	SAFA	MUFA	PUFA
1. Rice, parboiled, milled 130.00	0.20	0.11	0.28
2. Whole wheat flour (atta) 50.00	0.10	0.07	0.37
3. Raw cowpea seeds 70.00	0.23	0.07	0.38
4. Sesame oil 10.00	1.42	3.97	4.17
5. Rice bran oil 34.00	7.00	13.36	11.90
6. Rice bran 20.50	0.85	1.55	1.53
7. Flaxseed 22.50 (9.00 tsp powder)	0.82	1.69	6.46 (O_3_: 5.13)
8. Coconut oil 5.36 (as 16.00 g of meat)^†^	4.65	0.23	0.06
9. Milk 600.00	11.16	4.86	1.20
10. Fenugreek seeds 12.50	0.10	0.09	0.39 (O_3_: 0.14)
11. Olive oil 1.25	0.17	0.91	0.13
Total^‡^	26.70	26.91	26.87

The specific quantities of fat sources were determined by calculating the amount of each item needed to provide the required quantity of a particular type of fat (e.g., MUFA) for the trial menu, while also contributing other fat types. This was done by multiplying the reciprocal of the fraction of a specific fat type in a food item by the desired amount of that fat type. In other words, to obtain “Y” grams of a particular fat (e.g., MUFA) from 100.00 g of an oil that contains “X” grams of it, the formula used was: (100 ÷ X) × Y. To balance the total contributions of SAFA, MUFA, and PUFA to approximately 26.70 g each, and to ensure adequate intake of vitamin B12, milk, commonly consumed in Indian villages, was added to the combination. Flaxseeds (rather than flaxseed oil) were included as the main source of omega-3 fatty acids, due to their wider availability across India. Additionally, fenugreek seeds were incorporated for their traditionally recognized antidiabetic properties.

Nutritive assessment of the combination of fat sources

The above combination clearly presents SAFA, MUFA, and PUFA components in roughly equal amounts (26.70 g each), with minor variations of ±0.30 g due to the natural variability in oil composition and the rounding of oil quantities to whole or 0.25 g increments.

At this stage of formulation, analysis of the fat sources showed that, except for the oils present in flaxseed and fenugreek seeds, which contained only omega-6 fatty acids in their PUFA fractions, the combination provided a total of 21.60 g of omega-6 fatty acids (Table 1, Block B: I; Table 3) [6,12-19].

To maintain an omega-6 to omega-3 ratio of 4:1, the remaining portion of the PUFA compartment (26.87 g - 21.60 g = 5.27 g) needed to be supplied by omega-3 fatty acids. In the final formulation, this was achieved through 9.50 g of flaxseed oil contained in 22.50 g of flaxseed, which contributes 5.13 g of omega-3 and 1.33 g of omega-6 fatty acids within its 6.46 g PUFA fraction. An additional 0.14 g of omega-3 fatty acids was derived from 12.50 g of fenugreek seeds. Thus, the resulting omega-6 to omega-3 ratio in the PUFA compartment was 4:1. The flaxseed quantity was rounded up to 22.50 g to facilitate practical measurement in the kitchen, equivalent to approximately nine teaspoons of powdered flaxseed.

Interim nutritive evaluation 2

After incorporating the fat source combination, the remaining deficits stood at -45.68 g for carbohydrate and -8.63 g for protein, these being the unresolved shortfalls from the original cereal-pulse combination following the addition of the fat sources. These gaps were expected to be filled by the carbohydrate and protein content of the yet-to-be-added protective foods. Up to this point, the cereal-pulse and fat source combination had already contributed a substantial amount of dietary fiber (35.79 g), which is equivalent to 94.18% of the recommended daily intake (Table 1, Block A: II; Supplemental Material 1).

The inclusion of fat sources also led to a marked increase in the levels of thiamin, riboflavin, pyridoxine, pantothenic acid, vitamin B12, phosphorus, magnesium, selenium, and all essential amino acids, bringing these nutrients well above their daily adequacy levels. However, a few protective nutrients remained deficient. While vitamins A, D, and K showed some improvement, they still remained below recommended levels, as did iodine and fluoride, which continued at zero due to their absence in both the cereal-pulse and fat source combinations (Table 4; Supplemental Material 4).

**Table 4:** Protective nutrients and essential amino acids from 130.00 g rice, 50.00 g whole wheat flour (atta), 70.00 g cowpea seeds, and the combination of fat sources (as detailed in Table 3). *Values for vitamins E and K reflect the total oil fraction; all other values are derived from rice bran and coconut meat only. **Retinol. ^†^Nutrient levels elevated to meet daily requirements by the addition of fat sources. -No data available on the detectable amount in the mentioned quantity.

		Fat-soluble vitamins				Water-soluble vitamins								Minerals and trace elements															Essential amino acids (adult male, 70.00 kg)									
Food items	Nutrients	Vitamin A/beta-carotene (IU or mcg)	Vitamin D (mcg)	Vitamin E (mg)	Vitamin K (mcg)	Thiamin (B1) (mg)	Riboflavin (B2) (mg)	Niacin (B3) (mg)	Pyridoxine (B6) (mg)	Pantothenic acid (B5) (mg)	Folate (B9) (mcg)	Vitamin B12 (mcg)	Vitamin C (mg)	Calcium (mg)	Phosphorus (mg)	Magnesium (mg)	Sodium (mg)	Potassium (mg)	Iron (mg)	Iodine (mcg)	Fluoride (mg)	Zinc (mg)	Copper (mcg)	Chromium (mcg)	Selenium (mcg)	Molybdenum (mcg)	Manganese (mg)	Chloride (g)	Histidine (mg)	Isoleucine (mg)	Leucine (mg)	Lysine (mg)	Methionine (mg)	Phenylalanine (mg)	Tyrosine (mg)	Threonine (mg)	Tryptophan (mg)	Valine (mg)
Cereal-pulse combination		Beta-carotene 22.34	6.72	0.46	6.20	1.03	0.32	5.91	0.67	2.21	470.59	0.00	1.05	103.01	636.30	226.04	16.33	1,117.10	8.78	0.00	0.00	5.19	1,182.50	79.50	34.41	152.50	3.59	_	884.89	1,289.32	2,404.34	1,588.23	579.80	1,746.70	1,092.77	1,091.81	372.07	1,690.17
Sesame oil 10.00 g		_	_	0.14	1.40	_	_	_	_	_	_	_	_	_	_	_	_	_	_	_	_	_	_	_	_	_	_	_	_	_	_	_	_	_	_	_	_	_
*Rice bran oil 38.25 g (4.25 g from rice bran 20.50 g)		0.00	0.00	12.35	9.45	0.56	0.06	6.97	0.83	1.51	12.92	0.00	0.00	11.69	344.40	160.11	1.03	303.40	3.81	0.00	0.00	1.24	149.24	0.00	3.20	0.00	2.91	_	72.78	116.44	209.10	133.25	62.73	130.18	84.26	113.78	22.14	180.61
Flaxseed 22.50 g (9.00 tsp powder)		0.00	0.00	0.07	0.97	0.37	0.04	0.69	0.11	0.22	19.58	0.00	0.14	57.38	144.45	88.20	6.75	182.93	1.29	0.00	0.00	0.98	274.50	0.23	5.71	5.00	0.56	_	106.20	201.60	279.00	193.95	83.25	215.33	110.93	172.35	66.83	240.75
Coconut oil 5.36 g (as 16.00 g meat)		0.00	0.00	0.04	0.03	0.01	0.00	0.09	0.01	0.05	4.16	0.00	0.53	2.24	18.08	5.12	3.20	56.96	0.39	0.00	0.00	0.18	69.60	0.48	1.62	1.92	0.24	_	12.32	20.96	39.52	23.52	9.92	27.04	16.48	19.36	6.24	32.32
Milk 600.00 g (excluding added vitamin D)		**270.00	7.80	0.42	7.80	0.36	1.08	0.72	0.24	2.40	30.0	2.70	0.00	678.00	504.00	60.00	258.00	792.00	0.24	0.00	0.00	2.28	150.0	24.00	22.80	_	0.00	_	570.00	978.00	1,794.00	1,584.00	498.00	978.00	954.00	804.00	240.00	1,236.0
Fenugreek seeds 12.50 g		17.75	0.25	0.00	0.19	0.04	0.02	0.15	0.10	0.03	6.39	0.00	0.00	16.88	54.38	20.88	5.03	111.38	1.05	0.00	0.00	0.48	145.00	1.88	1.25	12.25	0.20	-	72.40	145.16	190.58	209.63	39.70	131.23	81.63	90.52	37.80	140.71
Olive oil 1.25 g		-	-	0.18	0.75	-	-	-	-	-	-	-	-	0.01	-	-	0.03	0.01	0.01	-	-	-	-	-	-	-	-	-	-	-	-	-	-	-	-	-	-	-
Total		Beta-carotene 40.09	14.77	13.66	26.79	2.37^†^	1.52^†^	14.53	1.96^†^	6.42^†^	543.64	2.70^†^	1.72	869.21	1,701.61^†^	560.35^†^	290.37	2,563.70	15.57	0.00	0.00	10.35	1,970.84	106.09	68.99^†^	171.67	7.50	_	1,718.59	2,751.48^†^	4,916.54^†^	3,732.58^†^	1,273.40^†^	3,228.48	2,340.07	2,291.82	745.08	3,520.56^†^
Remarks		Stored nutrient. Retinol 270.00	Stored nutrient	Stored nutrient	Stored nutrient	Adequate	Adequate	Deficit of 1.47 mg	Adequate	Adequate	Adequate	Stored nutrient. Adequate	Deficit of 88.28	Deficit of 130.79	Adequate	Adequate	4.4 g NaCl provides adequate amount	Deficit of 836.30 mg	Adequate	Tissue-stored nutrient	Adequate water levels in India	Deficit of 0.65 mg	Adequate	Adequate	Adequate	Adequate	Adequate	4.4 g NaCl provides adequate amount	Adequate	Adequate	Adequate	Adequate	Adequate for the combined ER of 1050.00	Adequate for the combined ER of 1750.00		Adequate	Adequate	Adequate

Selection and addition of protective food items to the model menu

At this stage, it became evident that the selection of protective food items for the menu had to address all remaining nutrient deficits without contributing significantly to additional fat intake. Since the formulation was intended for a specific geographic population with generally low income and distinct food customs, both local availability and traditional dietary patterns were taken into account. As such, the protective food items listed in Table 1 (Block A: III; Indian gooseberry, arrowroot flour, onion stalk, chili powder, drumstick leaves, ash gourd, fresh ginger, and raw guava) were selected [12,20-22]. An additional consideration was the inclusion of locally available bulk-forming foods commonly used in the Indian diet (e.g., ash gourd and drumstick leaves) to promote satiety ("hunger-killing") without significantly increasing caloric intake, thus helping prevent overconsumption of calorie-dense foods.

Interim nutritive evaluation 3

The protective food items listed in Table 1 (Block A: III) provided sufficient amounts of carbohydrate and protein (45.84 g and 8.66 g, respectively) to fully meet the final daily deficits remaining after the addition of the fat sources. The slight excess (45.84 g vs. 45.68 g of carbohydrate and 8.66 g vs. 8.63 g of protein) is clinically negligible. These food items also addressed the fiber shortfall and improved the levels of most protective nutrients, while contributing a minor excess of 1.84 g of fat (Tables 5 and 6; Supplemental Materials 5 and 6).

**Table 5:** Contributions of fat (invisible) from protective food items. Source: [12].

Protective food items contributing additional fats (invisible)	Additional fats
15. Chili powder 6.00 g	0.86 g
16. Drumstick leaves 60.00 g	0.98 g
Total of additional fats from protective food items	1.84 g

**Table 6:** Nutrients from protective food items.

		Fat-soluble vitamins				Water-soluble vitamins								Minerals and trace elements															
Food items	Nutrients	Beta-carotene (3,600 mcg/d)	Vitamin D (mcg)	Vitamin E (mg)	Vitamin K (mcg)	Thiamin (B1) (mg)	Riboflavin (B2) (mg)	Niacin (B3) (mg)	Pyridoxine (B6) (mg)	Pantothenic acid (B5) (mg)	Folate (B9) (mcg)	Vitamin B12 (mcg)	Vitamin C	Calcium (mg)	Phosphorus (mg)	Magnesium (mg)	Sodium(mg)	Potassium (mg)	Iron (mg)	Iodine (mcg)	Fluoride (mg)	Zinc (mg)	Copper (mcg)	Chromium (mcg)	Selenium (mcg)	Molybdenum (mcg)	Manganese (mg)	Chloride (g)	Remarks
Indian gooseberry 15.00 g		_	_	_	_	0.00	0.00	0.05	_	_	_	0.00	90.00	7.50	_	_	0.75	33.75	0.18	_	_	_	_	_	_	_	_	_	"-" means no data available on the detectable amount in the mentioned quantity
Arrowroot flour 35.50 g		_	_	_	_	0.00	0.00	0.00	0.00	0.05	2.49	0.00	0.00	14.20	1.78	1.07	0.71	3.91	0.12	_	_	0.02	14.20	_	_	_	0.17	_	
Onion stalk 125.00 g		875.00	8.51	1.01	55.41	0.04	0.06	0.18	0.21	0.24	72.01	0.00	34.03	38.90	35.66	83.39	19.40	390.00	3.86	0.00	0.00	1.24	175.00	73.75	6.53	21.25	0.28	_	
Chili powder 6.00 g		92.52	1.46	0.04	16.44	0.03	0.05	0.42	0.03	0.03	3.09	0.00	-	6.00	16.80	13.86	1.17	134.70	0.37	0.00	0.00	0.10	52.20	6.84	1.13	0.72	0.90	_	
Drumstick leaves 60.00 g		10,525.20	8.60	0.18	287.40	0.04	0.27	0.49	0.52	0.23	25.73	0.00	64.80	188.40	65.40	58.25	5.60	238.20	2.74	_	_	0.43	264.00	12.00	2.37	21.60	0.76	_	
Ash gourd 50.00 g		0.00	0.68	0.01	17.65	0.02	0.01	0.06	0.09	0.19	7.06	0.00	5.71	9.70	14.54	10.00	0.39	186.00	0.24	0.00	0.00	0.07	20.00	3.00	0.58	4.00	0.05	_	
Ginger raw, fresh 5.00 g		4.43	0.20	0.01	1.28	0.00	0.00	0.02	0.01	0.01	0.54	0.00	0.27	0.94	2.22	2.73	0.50	20.35	0.10	_	_	0.02	6.50	0.65	_	0.10	0.19	_	
Guavas 40.00 g (common, raw)		149.60	_	0.29	1.04	0.03	0.02	0.43	0.04	0.18	19.60	_	91.20	7.20	16.0	8.80	0.80	166.80	0.10	_	_	0.09	92.00	3.60	0.24	1.20	0.06	_	
Totals		Beta-carotene 12,046.75	19.45	1.54	379.22	0.16	0.41	1.65	0.90	0.93	130.52	0.00	286.01	272.84	152.40	178.10	29.32	1173.41	7.71	0.00	0.00	1.97	624.20	99.84	10.85	48.87	2.41	_	

## Results

This predominantly vegetarian menu formulation for the prototype balanced diet provided adequate amounts of proximate nutrients, essential amino acids, dietary fiber, and most key vitamins. However, it revealed partial deficiencies in certain protective nutrients, specifically sodium and chloride, and complete deficiencies in iodine and fluoride (Table 7) [23,24]. In total, the newly developed menu, designed to incorporate 43 nutrients (comprising 6 macronutrients (including specific fat components), 10 essential amino acids, and 27 protective nutrients), showed deficiencies in only four protective nutrients upon completion of the tabulation. The reasons for these deficiencies and potential solutions are discussed below. Importantly, none of the nutrients exceeded their respective "tolerable upper intake levels" (TULs; Supplemental Material 2) [25].

**Table 7:** Grand totals of protective nutrients from the cereal-pulse combination, the combination of fat sources, and the protective food items, as presented in Tables 2, 4, and 6. ^*^12,086.84 mcg beta-carotene and 270.00 mcg retinol. -No data available on the detectable amount in the mentioned quantity. TUL: tolerable upper intake level.

	Fat-soluble vitamins				Water-soluble vitamins								Minerals and trace elements														
Nutrients	Vitamin A (IU or mcg)	Vitamin D (mcg)	Vitamin E (mg)	Vitamin K(mcg)	Thiamin (B1) (mg)	Riboflavin (B2) (mg)	Niacin (B3) (mg)	Pyridoxine (B6) (mg)	Pantothenic acid (B5) (mg)	Folate (B9) (mcg)	Vitamin B12 (mcg)	Vitamin C (mg)	Calcium (mg)	Phosphorus (mg)	Magnesium (mg)	Sodium (mg)	Potassium (mg)	Iron (mg)	Iodine (mcg)	Fluoride (mg)	Zinc (mg)	Copper (mcg)	*Chromium (mcg)	Selenium (mcg)	*Molybdenum (mcg)	Manganese (mg)	Chloride (g)
Adult: Recommended dietary allowance (RDA), adequate intake (AI), or estimated average requirement (EAR) per day	RDA: 900 mcg (beta-carotene 3,600 mcg)	RDA: 15	RDA: 15	AI: 120	RDA: 1.2	RDA: 1.3	RDA: 16	RDA: 1.3	AI: 5.0	RDA: 400	RDA: 2.4	RDA: 90	RDA: 1,000	RDA: 700	RDA: 400	AI: 1,500	AI: 3,400	RDA: 8.0	RDA: 150	AI: 4.0	RDA: 11	RDA: 900	AI: 35	RDA: 55	RDA: 45	AI: 2.3	AI: 2.3
Food items	Retinol 270.00 (beta-carotene 40.09)	14.77	13.66	26.79	2.37	1.52	14.53	1.96	6.42	543.64	2.70	1.72	869.21	1,701.61	560.35	290.37	2,563.70	15.57	0.00	0.00	10.35	1,970.84	106.09	68.99	171.67	7.50	_
Acquired from Tables 2 and 4 (rice, wheat, cowpea seeds, and fat sources)																											
Acquired from Table 6 (protective food items)	Beta-carotene 12,046.75	19.45	1.54	379.22	0.16	0.41	1.65	0.90	0.93	130.52	0.00	286.01	272.84	152.40	178.10	29.32	1,173.41	7.71	0.00	0.00	1.97	624.20	99.84	10.85	48.87	2.41	-
Total gain from both categories (surplus amount, for the anticipated cooking loss)	*12,086.84 (+8,684.84)	34.22 (+19.22)	15.20 (+0.20)	406.01 (+286.01)	2.53 (+1.33)	1.93 (+0.63)	16.18 (+0.18)	2.86 (+1.56)	7.35 (+2.35)	674.16 (+274.16)	2.70 (+0.30)	287.73 (+197.73)	1,142.05 (+142.05)	1,854.01 (+1,154.01)	738.45 (+338.45)	319.69 (-1,180.31)	3,737.11 (+337.11)	23.28 (+15.28)	0.00	0.00	12.32 (+1.32)	2,595.04 (+1,695.04)	205.93 (+170.93)	79.84 (+24.84)	220.54 (+175.54)	9.91 (+7.61)	0.00 (-2.3)
Remarks (tolerable upper intake levels (TULs) [12])	Stored nutrient. Adequate*	Stored nutrient. Adequate	Stored nutrient. Adequate	Stored nutrient. Adequate	Adequate	Adequate	Adequate	Adequate	Adequate	Adequate	Stored nutrient. Adequate	Adequate	Adequate and below TUL	Adequate and below TUL	Adequate	4.4 g NaCl provides adequate amount	Adequate	Adequate and below TUL	Tissue-stored nutrient	In India, water levels are adequate	Adequate and below TUL	Adequate and below TUL	Adequate and TUL not known* [12]	Adequate and below TUL	Adequate and below TUL* [12]	Adequate and below TUL	4.4 g NaCl provides adequate amount

Deficiencies of sodium and chloride can be managed through the currently recommended minimum daily intake of table salt, 4.40 g/day (75.00 meq) [26].

Daily intake of body-stored nutrients such as fat-soluble vitamins and vitamin B12 is not strictly required. Nonetheless, this formulation provides adequate levels of vitamins A, D, E, and K. Notably, the region’s nearly year-round sun exposure naturally supports sufficient vitamin D synthesis. Among the included food items, milk alone accounts for the full daily vitamin B12 requirement (2.40 mcg), and it remains a staple in the diet of Indian village farmers, who form a major part of the population.

Fluoride and iodine, also body-stored nutrients, remain deficient in the new menu. Fluoride is generally not obtained through food, and iodine availability in vegetables varies depending on the iodine content of the soil, which is higher in coastal regions. Due to these factors, reliable generalized nutritive data for these nutrients are not available (from both cited and additional reference sources), making it difficult to estimate their intake from this menu. 

We therefore recommend the regular use of iodized salt as the primary source of dietary iodine. India’s public health infrastructure ensures its widespread availability. Alternatively, including sea products in the diet, along with corresponding adjustments to the food list and nutrient tabulation, can help meet iodine needs.

Fluoride intake, on the other hand, mainly comes from drinking water. In most parts of India, fluoride levels in water are around 0.50 mg/L, which is generally sufficient for preventing deficiency, especially given the higher water consumption typical in tropical regions [27]. In areas with low fluoride levels in water, supplementation through water fluoridation, fluoridated foods, toothpastes, or other means is recommended.

Two other protective nutrients, chromium and molybdenum, require special mention. Data for these were obtained from a separate source [12], as earlier references lacked this information. Based on this source, the current menu meets recommended intake levels for both nutrients, well within their TULs.

Lastly, a small amount of additional fat (1.84 g, or 1.50 g depending on the source) came from the protective food items (Table 5). However, due to its minimal quantity, it was not included in the initial fat combination structure (Table 1, Block B: II), as it was too minor to affect the formulation.

## Discussion

A non-diabetic adult requiring 2,000 calories per day typically derives 50%-60% of these calories from carbohydrates, 10%-14% from proteins, and the remainder from fats, without exceeding 30% (or 80 g) of total calories from fat [20,24,28]. In diabetes mellitus, although the cellular mechanism for energy extraction from carbohydrates is impaired, the overall daily energy requirement remains unchanged. Therefore, the most appropriate nutritional strategy is to reduce the carbohydrate load while proportionally increasing protein and fat intake to meet energy needs. This principle is already reflected in traditional diabetic diets such as the TMD and the ADA sample plans. However, similar dietetic models using routine Indian food items remained largely conceptual. It was in this context, where both standardized menu plans and formulation guidelines for Indian diets were lacking, that the authors designed the present study. The proposed work introduces a model menu formulated with mathematically predicted nutrient values, using traditional Indian foods in culturally typical quantities. Beyond serving as a prototype, this formulation offers a practical framework for developing other metabolically targeted diets. It also helps reduce the cost and inefficiency of clinical trials by minimizing reliance on random or poorly planned dietary models. Since this menu is a nutritionally equivalent adaptation of well-established models like TMD and ADA, it can be clinically evaluated for its therapeutic value upon ethical committee approval and laboratory confirmation of the calculated nutrient contents. Furthermore, the formulation methodology and its accompanying interim evaluation tables can serve as nutritional assessment tools for both traditional and newly designed diet plans, before and after actual recipe preparation.

The source recommending the consumption of equal amounts of SAFA, MUFA, and PUFA is the Dietary Goals for the United States, 1977 [7]. This guideline was selected for constructing the new menu because it was the first to clearly suggest the proportionate intake of each type of fat, following the initial dietary recommendations introduced by the American Heart Association in 1957 [29]. Technically, to translate these fat requirements into numerical values, i.e., the specific amounts of SAFA, MUFA, and PUFA, explicit recommendations on the percentage requirement of each component are essential. However, both earlier and later dietary guidelines have generally lacked such clarity, particularly regarding MUFA and PUFA requirements. This absence of detailed guidance makes it challenging to advise or prescribe specific menus for patients [30]. Possibly due to this technical clarity, many medical textbooks and educators during the 1980s and 1990s referred to the 1977 recommendations as the “classic” guidelines. These continue to be highlighted, especially when paired with the later-established 4:1 ratio between omega-6 and omega-3 fatty acids, together often cited as the “classic recommendations” for dietary fat consumption.

The term “omega ratio” is a conventional teaching expression commonly used in medical classrooms to refer to the ratio between omega-6 and omega-3 fatty acids. Although informal, it was included in this article for the convenience it offers in making repeated references during discussions on dietary fats.

The menu formulation developed in this study is specifically designed to meet the caloric and protective nutrient needs of a diabetic adult with minimal physical exertion (i.e., a 2,000-calorie requirement). For other individuals, such as diabetic adults with higher energy demands, the energy distribution percentages (from carbohydrates, proteins, and fats) may need adjustment based on the intensity and type of physical activity (e.g., brisk versus sustained exertion) before applying this methodology. In cases of comorbid conditions (such as liver disease), both the daily caloric target and the choice of food items may also require appropriate modification.

Finally, it is important to remember that the selection of food items in menu formulations must be adjusted to account for quantitative and qualitative variations in nutritional requirements among diabetic individuals, based on factors such as age, sex, and physiological status (e.g., pregnancy). These considerations are equally relevant to the non-diabetic population.

Since this study is predictive in nature, the projected nutritional values must be validated through post-cooking analysis of the prepared recipes. If any nutrient, particularly those not stored in the body, is found to be below the required daily level, a step-by-step reassessment should be carried out. This may involve examining cooking methods, post-harvest storage practices, or pre-cooking nutrient content of ingredients. Any identified issue should be corrected to ensure nutritional adequacy before initiating clinical dietary trials using this or any similar predictive menu formulation.

## Conclusions

From a nutritional perspective, the most logical way to manage the metabolic condition of diabetes mellitus is by rearranging the pattern of energy derivation, specifically, through a controlled reduction in carbohydrate intake and compensatory increases in protein and fat intake. In line with this approach, the authors have successfully proposed a target-oriented menu formulation composed primarily of low-glycemic-index local Indian food items (G.I. < 55), using a straightforward methodology to support the overall well-being of diabetic adults. Notably, in this formulation, the carbohydrate-derived energy from rice and wheat, the “key foods,” does not dominate the total carbohydrate share but instead contributes nearly equally with the rest of the items, at 26.48% and 23.52%, respectively. The methodology is flexible enough to design metabolically targeted menu formulations tailored to various physiological and clinical needs, enabling diverse food cultures worldwide to adopt contextually balanced diets using locally available foods, while preserving traditional tastes and minimizing costs.

Importantly, this is the first instance in diabetic nutrition where a complete balanced diet has been developed using everyday Indian food items, with carbohydrate-derived energy restricted to 50%. The inclusion of fat components derived in precise proportions from constituent food items also introduces a novel concept to diabetic dietary planning.

## Appendices

Supplemental material 1

**Table 8:** Carbohydrate, protein, fat, and dietary fiber content per 100.00 g of each food item. SAFA, saturated fatty acid, MUFA: monounsaturated fatty acid, PUFA: polyunsaturated fatty acid, O_6_: omega-6 acid, O_3_: omega-3 acid. *Total fat content of items 1, 2, and 3 (in 100.00 g):  0.55 + 1.53 + 1.26 = 3.34 g. Source:  [6,12-22].

Food items	Block A			Block B		
	CHO (g) (excluding fiber)	Protein (g)	Fiber (g)	SAFA (g)	MUFA (g)	PUFA (g)
I. Cereal-pulse block				*I. The initial fat combination		
1. Rice, parboiled, milled 100.00 g	77.16	7.81	3.74	0.15	0.09	0.21
2. Wheat whole flour (atta) 100.00 g	64.17	10.57	11.36	0.21	0.15	0.74
3. Raw cowpea seeds 100 g	49.40	23.50	10.60	0.33	0.11	0.54
II. Fat/oil block						
4. Sesame oil 100.00 g				14.20	39.70	41.70
5. Rice bran oil 100.00 g				19.70	39.30	35.00
6. Rice bran, crude 100.00 g	28.70	13.40	21.00	4.17	7.55	7.46
7. Flaxseed 100.00 g (40.00 tsp powder)	1.60	18.30	27.30	3.66	7.53	28.70 (O_3_: 22.80)
8. Coconut meat, fresh 100.00 g (with 33.50 g oil)	6.20	3.33	9.00	29.07	1.42	0.37
9. Milk 100.00 g	4.80	3.15	0.00	1.86	0.81	0.20
10. Fenugreek seeds 100.00 g	10.57	25.41	47.55	0.77	0.68	3.13 (O_3_: 1.08)
11. Olive oil 100.00 g				13.81	72.96	10.52
III. Protectives’ block				II. Details of fat from the protective food items		
12. Indian gooseberry 100.00 g	13.70	0.50	3.40			
13. Arrowroot flour 100.00 g	84.80	0.30	3.40			
14. Onion stalk 100.00 g	2.49	1.86	5.10			
15. Chili powder, red 100.00 g	29.46	12.69	31.15	1.14	7.18	3.26 (O_6_: 3.08; O_3_: 0.18)
16. Drumstick leaves 100.00 g	5.62	6.41	8.21	0.56	0.10	0.67 (O_6_: 0.22; O_3_: 0.45)
17. Ash gourd 100.00 g	2.84	0.79	3.37			
18. Ginger raw 100.00 g	8.97	2.22	5.36			
19. Guavas, common, raw 100.00 g	8.90	2.55	5.40			

Supplemental material 2 

**Table 9:** Protective nutrients and essential amino acids in 100.00 g of rice, whole wheat flour, and raw mature cowpea seeds, alongside their tolerable upper intake levels.

	Fat-soluble vitamins				Water-soluble vitamins								Minerals and trace elements															Essential amino acids									
Nutrients	Vitamin A/beta-carotene (IU or mcg)	Vitamin D (mcg)	Vitamin E (mg)	Vitamin K (mcg)	Thiamin (B1) (mg)	Riboflavin (B2) (mg)	Niacin (B3) (mg)	Pyridoxine (B6) (mg)	Pantothenic acid (B5) (mg)	Folate (B9) (mcg)	Vitamin B12 (mcg)	Vitamin C (mg)	Calcium (mg)	Phosphorus (mg)	Magnesium (mg)	Sodium (mg)	Potassium (mg)	Iron (mg)	Iodine (mcg)	Fluoride (mg)	Zinc (mg)	Copper (mcg)	Chromium (mcg) [12]	Selenium (mcg)	Molybdenum (mcg)[12]	Manganese (mg)	Chloride (g)	Histidine (mg)	Isoleucine (mg)	Leucine (mg)	Lysine (mg)	Methionine (mg)	Phenylalanine (mg)	Tyrosine (mg)	Threonine (mg)	Tryptophan (mg)	Valine (mg)
Food items																																					
Rice 100.00 g	0	0	0.06	1.5	0.17	0.06	2.51	0.22	0.55	9.75	0	0	8.11	140	26.72	3.16	142	0.72	0	0	1.08	270	5	1.19	55	0.79	_	183.53	323.33	631.05	267.1	193.69	401.43	345.98	253.04	89.81	488.91
Wheat flour, whole 100.00 g (atta)	2.67	13.43	0.21	1.5	0.42	0.15	2.37	0.25	0.87	29.22	0	0	30.94	315	125	2.04	311	4.1	0	0	2.85	480	6	53.12	22	2.98		270.59	399.55	647.94	255.79	187.09	531.67	221.97	272.71	104.64	541.18
Raw cowpea seeds 100.00 g	30	0	0.39	5	0.85	0.23	2.08	0.36	1.5	633	0	1.5	110	424	184..00	16	1110	8.27	0	0	3.37	845	100	9	100	1.53	_	730	956	1800	1590	335	1370	760	895	290	1120
Tolerable upper intake levels (TULs) [25]													2,500 mg/day	4,000 mg/day				45 mg/day	1,100 mcg/day	10 mg/day	40 mg/day	10,000 mcg/day	Not determined	400 mcg/day	2,000 mcg/day	11 mg/day											

Supplemental material 3 

**Table 10:** Saturated, monounsaturated, and polyunsaturated fatty acids in 100.00 g of constituent dietary fat sources (combined fat sources). SAFA: saturated fatty acid, MUFA: monounsaturated fatty acid, PUFA: polyunsaturated fatty acid, O_3_: omega-3 fatty acid. Source: [6,12-19].

Food items (g)	Fatty acids (g)		
	SAFA	MUFA	PUFA
1. Rice, parboiled, milled 100.00	0.15	0.09	0.21
2. Whole wheat flour (atta) 100.00	0.21	0.15	0.74
3. Raw cowpea seeds 100.00	0.33	0.11	0.54
4. Sesame oil 100.00	14.20	39.70	41.70
5. Rice bran oil 100.00	19.70	39.30	35.00
6. Rice bran 100.50	4.17	7.55	7.46
7. Flaxseed 100.00 (40.00 tsp powder)	3.66	7.53	28.70 (O_3_: 22.80)
8. Coconut oil 100.00 (100.00 g fresh coconut meat - 33.50 g oil only)	29.07	1.42	0.37
9. Milk 100.00	1.86	0.81	0.20
10. Fenugreek seeds 100.00	0.77	0.68	3.13 (O_3_: 1.08)
11. Olive oil 100.00	13.81	72.96	10.52

Supplemental material 4 

**Table 11:** Protective nutrients and essential amino acids in 100.00 g of constituent dietary fat sources. *Retinol.

		Vitamins A, D, E, and K					Water-soluble vitamins											Minerals and trace elements																					Essential amino acids									
Food items	Nutrients	Vitamin A/beta-carotene (IU or mcg)	Vitamin D (mcg)	Vitamin E (mg)		Vitamin K (mcg)	Thiamin (B1) (mg)	Riboflavin (B2) (mg)	Niacin (B3) (mg)		Pyridoxine (B6) (mg)		Pantothenic acid (B5) (mg)	Folate (B9) (mcg)	Vitamin B12 (mcg)		Vitamin C (mg)	Calcium (mg)	Phosphorus (mg)	Magnesium (mg)		Sodium (mg)	Potassium (mg)		Iron (mg)		Iodine (mcg)	Fluoride (mg)		Zinc (mg)		Copper (mcg)	Chromium (mcg)[12]		Selenium (mcg)	Molybdenum (mcg)[12]	Manganese (mg)	Chloride (g)	Histidine (mg)	Isoleucine (mg)	Leucine (mg)	Lysine (mg)	Methionine (mg)	Phenylalanine (mg)	Tyrosine (mg)	Threonine (mg)	Tryptophan (mg)	Valine (mg)
																																																Sesame oil 100.00 g		-	-	1.40	13.60		_	_	_	_		_		_	_	_		_	_	_	_		_	_		_		_	_		_		_	_		_	_	_	_	_	_	_	_	_	_	_	_	_
Rice bran oil 100.00 g		_	_	32.30	24.70		_	_	_	_		_		_	_	_		_	_	_	_		_	0.07		_		_	_		_		_	_		_	_	_	_	_	_-	_	_	_	_	_	_	_
Rice bran, crude 100.00 g		0.00	0.00	4.92	1.90		2.75	0.28	34.00	4.07		7.39		63.00	0.00	0.00		57.00	1,680.00	781.00	5.00		1,480.00	18.50		-		-	6.04		728.00		-	15.60		-	14.20		355.00	568.00	1,020.00	650.00	306.00	635.00	411.00	555.00	108.00	881.00
Flaxseed 100.00 g		0.00	0.00	0.31	4.33		1.64	0.16	3.08	0.47		0.99		87.00	-	0.60		255.00	642.00	392.00	30.00		813.00	5.73		00.0		00.0	4.34		1,220.00		1.00	25.40		22.00	2.48	_	472.00	896.00	1,240.00	862.00	370.00	957.00	493.00	766.00	297.00	1070.00
Coconut meat, fresh 100.00 g		0.00	0.00	0.24	0.20		0.07	0.02	0.54	0.05		0.30		26.00	0.00	3.30		14.00	113.00	32.00	20.00		356.00	2.43		0.00		0.00	1.10		435.00		3.00	10.10		12.00	1.50	_	77.00	131.00	247.00	147.00	62.00	169.00	103.00	121.00	39.00	202.00
Milk 100.00 g (excluding added vitamin D)		45.00*	1.30	0.07	1.30		0.06	0.18	0.12	0.04		0.40		5.00	0.45	0.00		113.00	84.00	10.0	43.0		132.0	0.04		0.00		0.00	0.38		25.00		4.00	3.80		_	0.00	_	95.00	163.00	299.00	264.00	83.00	163.00	159.00	134.00	40.00	206.00
Fenugreek seeds 100.00g		142.00	1.98	0.02	1.50		0.28	0.14	1.19	0.77		0.27		51.11	0.00	0.00		135.00	435.00	167.00	40.20		891.00	8.47		0.00		0.00	3.80		1160.00		15.00	9.98		98.00	1.60	-	579.35	1161.24	1524.60	1677.06	317.63	1049.81	653.04	724.19	302.38	1125.66
Olive oil 100.00 g		_	_	14.40	60.20		_	_	_	_		_		_	_	_		1.00	_	_	2.00		1.00	0.56		_		_	_		_		_	_		_	_	_	_	_	_	_	_	_	_	_	_	_

Supplemental material 5 

**Table 12:** Invisible fat content in 100.00 g of protective food items. Source: [12].

Protective food items contributing additional fat (invisible)	Additional fat
15. Chili powder 100.00 g	14.30 g
16. Drumstick leaves 100.00 g	1.64 g

Supplemental material 6 

**Table 13:** Protective nutrients in 100.00 g of protective food items.

		Fat-soluble vitamins				Water-soluble vitamins								Minerals and trace elements															
Food items	Nutrients	Beta-carotene (3,600 mcg/d)	Vitamin D (mcg)	Vitamin E (mg)	Vitamin K (mcg)	Thiamin (B1) (mg)	Riboflavin (B2) (mg)	Niacin (B3) (mg)	Pyridoxine (B6) (mg)	Pantothenic acid (B5) (mg)	Folate (B9) (mcg)	Vitamin B12 (mcg)	Vitamin C	Calcium (mg)	Phosphorus (mg)	Magnesium (mg)	Sodium(mg)	Potassium (mg)	Iron (mg)	Iodine (mcg)	Fluoride (mg)	Zinc (mg)	Copper (mcg)	Chromium (mcg)[12]	Selenium (mcg)	Molybdenum (mcg) [12]	Manganese (mg)	Chloride (g)	Remarks
Indian gooseberry 100.00 g		_	_	_	_	0.03	0.01	0.20	_	_	_	_	600.00	50.00	_	_	5.00	225.00	1.20	_	_	_	_	_	_	_	_	_	NB: "-" means no data available on the detectable amount in the mentioned quantity
Arrowroot flour 100.00 g		0.00	0.00	0.00	0.00	0.00	0.00	0.00	0.01	0.13	7.00	__	-	40.00	5.00	3.00	2.00	11.00	0.33	-	-	0.07	40.00	__	__	__	0.47	-	
Onion stalk 100.00 g		700.00	6.81	0.81	44.33	0.03	0.05	0.14	0.17	0.19	57.61	-	27.23	31.12	28.53	66.71	15.52	312.00	3.09	-	-	0.99	140.00	59.00	5.22	17.00	0.22	-	
Chili powder, red 100.00 g		1,542.00	24.36	0.69	274.00	0.46	0.83	6.94	0.42	0.57	51.50	0.00	-	99.83	280.00	231.00	19.45	2,245.00	6.23	0.00	0.00	1.66	870.00	114.00	18.83	12.00	1.45	__	
Drumstick leaves 100.00 g		17,542.00	14.33	0.30	479.00	0.06	0.45	0.82	0.87	0.39	42.89	0.00	108.00	314.00	109.00	97.09	9.34	397.00	4.56	0.00	0.00	0.72	440.00	20.00	3.95	36.00	1.26		
Ash gourd 100.00 g		0.00	1.35	0.01	35.30	0.03	0.01	0.12	0.18	0.37	14.11	0.00	11.41	19.39	29.07	19.95	0.77	372.00	0.47	0.00	0.00	0.13	40.00	6.00	1.15	8.00	0.09	__	
Ginger raw, fresh 100.00 g		88.85	4.09	0.28	25.55	0.04	0.04	0.42	0.20	0.24	10.82	0.00	5.43	18.88	44.36	54.66	10.03	407.00	1.90	_	_	0.39	130.00	13.00	-	2.00	3.85	__	
Guavas, common, raw 100.00 g		374.00	0.00	0.73	2.60	0.07	0.04	1.08	0.11	0.45	49.00	0.00	228.0	18.00	40.00	22.00	2.00	417.00	0.26	-	-	0.23	230.00	9.00	0.60	3.00	0.15	-	
